# Micronutrient deficiency in obese subjects undergoing low calorie diet

**DOI:** 10.1186/1475-2891-11-34

**Published:** 2012-06-01

**Authors:** Antje Damms-Machado, Gesine Weser, Stephan C Bischoff

**Affiliations:** 1 Department of Nutritional Medicine, University of Hohenheim, Stuttgart, Germany; 2 Department of Nutritional Medicine, University of Hohenheim, Fruwirthstr. 12, ss70599, Stuttgart, Germany

**Keywords:** Obesity, Low calorie diet, Weight loss, Micronutrient deficiency, Dietary reference intake

## Abstract

**Background:**

The prevalence of micronutrient deficiencies is higher in obese individuals compared to normal-weight people, probably because of inadequate eating habits but also due to increased demands among overweight persons, which are underestimated by dietary reference intakes (DRI) intended for the general population. We therefore evaluated the dietary micronutrient intake in obese individuals compared to a reference population and DRI recommendations. Furthermore, we determined the micronutrient status in obese subjects undergoing a standardized DRI-covering low-calorie formula diet to analyze if the DRI meet the micronutrient requirements of obese individuals.

**Methods:**

In 104 subjects baseline micronutrient intake was determined by dietary record collection. A randomly assigned subgroup of subjects (n = 32) underwent a standardized DRI-covering low-calorie formula diet over a period of three months. Pre- and post-interventional intracellular micronutrient status in buccal mucosa cells (BMC) was analyzed, as well as additional micronutrient serum concentrations in 14 of the subjects.

**Results:**

Prior to dietetic intervention, nutrition was calorie-rich and micronutrient-poor. Baseline deficiencies in serum concentrations were observed for 25-hydroxyvitamin-D, vitamin C, selenium, iron, as well as ß-carotene, vitamin C, and lycopene in BMC. After a three-month period of formula diet even more subjects had reduced micronutrient levels of vitamin C (serum, BMC), zinc, and lycopene. There was a significant negative correlation between lipophilic serum vitamin concentrations and body fat, as well as between iron and C-reactive protein.

**Conclusions:**

The present pilot study shows that micronutrient deficiency occurring in obese individuals is not corrected by protein-rich formula diet containing vitamins and minerals according to DRI. In contrast, micronutrient levels remain low or become even lower, which might be explained by insufficient intake, increased demand and unbalanced dispersal of lipophilic compounds in the body.

**Trial registration:**

The study was registered at ClinicalTrials.gov (NCT01344525). The study protocol comprises only a part of the approved trial protocol.

## Background

In general, occurrence of malnutrition is thought to be disease-related and/or associated with undernourishment. During the past few years, evidence has been raised that obesity can also be associated with substantial nutrient deficiencies [1-5]. In fact, the prevalence of micronutrient deficiencies in obese individuals is higher compared to normal weight controls of the same age and sex [6,7], and affects several micronutrients such as zinc [4,8], selenium [4,6,8,9], folate [4,6,8,10], vitamin B_1_[11], vitamin B_12_[4,6,8,10,12], vitamin A [6,8], vitamin E [4,6], and 25-hydroxyvitamin D [25(OH)D] [4,6,8,11]. Imbalances or deficiencies of essential micronutrients significantly influence day-to-day performance, behavior and emotional state, as well as intellectual and physical activity [13-15]. It has been hypothesized that toxic by-products of incomplete biochemical reactions resulting from excessive intake of kilocalories and states of micronutrient deficiencies could lead to further weight gain or development of associated metabolic diseases [13,16]. Considering the worldwide prevalence of obesity, a significant part of the population could be afflicted by this type of micronutrient deficiency, even in wealthy Western countries.

The dietary reference intakes (DRI) for daily supply of vitamins and mineral nutrients apply to healthy, normal weight individuals. They account for individual variations and distinguish between age groups and sex [17,18], but they do not apply to patients with metabolic alterations or other disease, or individuals using pharmaceuticals on a regular basis. Hence, DRI do not necessarily meet the metabolic needs of obese individuals. Especially in physiologic stress situations like significant weight loss or periods of weight cycling, obese patients potentially need different amounts of micronutrients.

The first aim of this pilot study was to investigate the micronutrient intake in obese subjects compared to a reference population and DRI recommendations. Second aim was to determine both serum and intracellular micronutrient status after a standardized DRI-covering low-calorie formula diet over three months in order to analyze if DRI for micronutrients apply to obese patients in a period of major weight loss. This setting was chosen, because it offers a unique possibility to study this issue using an entirely standardized diet consumed by high-grade obese individuals over a longer-term period.

## Methods

### Intervention and study population

This pilot study included obese individuals participating in a multidisciplinary weight loss program (OPTIFAST®52) causing an average weight loss of 26.0 kg in men and 19.6 kg in women within one year [19]. Briefly, the program consists of a five-phase lifestyle modification program designed for 52 weeks, including (i) a 1-week introduction during which a detailed nutrition analysis is performed; (ii) a 12 week period of low-calorie diet (LCD) (800 kcal/day) during which participants consume a formula diet exclusively (daily consumption of 5 sachets at 160 kcal each, Optifast 800® formula, Nestlé Inc.); (iii) a 6 weeks refeeding phase, (iv) a 7 weeks stabilization phase and (v) a 26 weeks maintenance phase. We previously reported more detailed information about the program [19]. The five daily consumed formula meals in the second phase contain vitamins, minerals and trace elements according to DRI for healthy adults (Table 1), except for the flavor potato/leek, which contains less amounts of vitamin C, vitamin B_12_, folate and calcium. However, flavor selection was documented, recording that the latter one was only consumed occasionally.

**Table 1:** Content of selected micronutrients in the formula diet and comparison with DRI (D-A-CH reference values)

**Micronutrient**	**Unit**	**Per sachet ^flavor A^ (42 g)**	% **of DRI per 5 sachets**	**Per sachet ^flavor B^ (42 g)**	% **of DRI per 5 sachets**
Vitamin A	mg	0.3	*♀*188; *♂*150	0.2	*♀*125; *♂*100
Vitamin D	μg	1.0	101	1.2	118
Vitamin E	mg	4.0	*♀*142; *♂*166	3.4	*♀*122; *♂*143
Vitamin C	mg	20.0	100	10.8	54
Vitamin B_12_	μg	0.6	98	0.4	60
Folate	μg	80.2	100	48.0	60
Calcium	mg	290.6	145	168.1	84
Iron	mg	4.0	*♂*133; *♀*199	3.8	*♂*127; *♀*191
Zinc	mg	3.0	*♂*149; *♀*213	2.3	*♂*114; *♀*163
Selenium	μg	20.2	144-336	13.6	97-227

*Flavor A*: vanilla, chocolate, strawberry, coffee, and tomato; *flavor B*: potato/leek.

To determine micronutrient intake in obese individuals we analyzed dietary record data obtained during the introduction week from all participants enrolled from 02/2006 to 02/2010 (n = 104). A randomly assigned subgroup of subjects (n = 32) participated in a pilot study, and was followed up for micronutrient measurement before and after the three-month formula diet period (LCD intervention group). Patients with a history of bariatric surgery were excluded from study participation. The measurements were performed by intracellular micronutrient analysis in buccal mucosa cells (BMC) and by additional serum micronutrient analysis in 14 of the subjects. The latter subjects did not differ in initial body weight and weight loss to the whole LCD intervention group. For 9 subjects, long-term data on intracellular vitamin concentrations were also obtained at the end of the program year, 9 months after completion of the LCD phase. The nutrients evaluated in this study were vitamin C (BMC, serum, leukocytes), vitamin E (BMC, serum), lycopene (BMC), β-carotene (BMC), vitamin A, 25(OH)D, vitamin B_12_, folate, selenium, iron, zinc and calcium (all serum). Deficiency was defined as a concentration below the reference interval: vitamin A 0.2-1.2 mg/ml, vitamin E 5–16 mg/ml (serum)/9.5-20.3 pmol/μg DNA (BMC), 25(OH)D, 20–70 ng/ml, vitamin C 28.3-85.1 μg/ml (serum)/57–114 nmol/10^8^ cells (leukocytes)/3.9-11.1 pmol/μg DNA (BMC), vitamin B_12_ 191–663 pg/ml, folate 4.6-18.7 ng/ml, selenium 74–139 μg/ml, iron 40–170 μg/dl, zinc 70–150 μg/dl, calcium 2.2-2.6 mmol/l, β-carotene 0.1-05 pmol/μg DNA, and lycopene 0.1-05 pmol/μg DNA. Moreover, C-reactive protein (CRP) concentrations were analyzed in serum samples. Drug use was monitored and documented on standardized forms. We calculated body mass index (BMI) and relative weight loss in percent (RWL; = 100xΔweight loss in kg/initial body weight in kg). Body composition was analyzed using bioelectrical impedance analysis at all study visits (Data Input Nutriguard M, Darmstadt, Germany). The study protocol was part of a multicenter clinical trial, research project “Obesity and the gastrointestinal tract” (ClinicalTrials.gov identifier: NCT01344525), approved by the ethics committee of the University Hospital of Tübingen, Germany. Informed consent was obtained from every subject prior to participation.

### Dietary records

Food intake was recorded using a predesigned daily journal. Subjects had to document time, amount (in gram) and situation, in which a food or beverage was consumed. They were instructed to: (1) document all consumed foods and beverages in as much detail as possible, (2) to weigh foods (or to estimate doses, if weighing was not possible in some situations), (3) to document food or beverage intake immediately after consumption and (4) not to change usual eating habits. Data analysis was performed using the nutrition software EBISpro, version 8.0 for Windows. In analyzing the eating records, nutrients were studied in relation to the DRI (D-A-CH reference values) [18]. The DRI utilized corresponds to the age group and sex. Furthermore, data were compared with the Second National Nutrition Survey (NNSII), which is representative for the German population. The dietary assessment in this survey was performed using controlled interviews according to the diet history method, which were reinforced by dietary record collection and 24 hour-recalls in a representative sub-sample.

### Intracellular micronutrient concentrations in buccal mucosa cells (BMC)

BMC were collected using a kit from Day-med-concept GmbH, Berlin, Germany. Subjects first had to rinse their mouths with water thoroughly to remove food particles and then brush the inner lining of their cheeks with a soft toothbrush twenty times, twice on each side. The toothbrush was washed in 25 ml NaCl solution (0.9%) gently after each brushing. The samples were then centrifuged at 1,600 rpm for 3 minutes. The supernatant was discarded; the cells were completely resuspended in rinsing solution (phosphor float 0.15% w/v) and centrifuged again at 1,800 rpm for 3 minutes. After removal of the supernatant fraction, the stabilizing solution (heat-sensitive reducing agent 0.09% w/v) was added; and the cells were resuspended and stored. The micronutrients in BMC (pmol/μg DNA) of the samples were measured by BioTeSys GmbH (Esslingen, Germany) using an accredited RP-HPLC method according to DIN EN ISO/IEC 17025. The reference values are based on a statistical distribution reflecting the 25th and 75th percentile of a data set covering analysed BMC samples over one year.

Micronutrient concentrations were expressed in pmol/μg DNA. Micronutrient detection was possible when the amount of DNA in BMC was at least sufficient (>1 μg). If the amount of DNA extracted was < 1 μg, the data was excluded from analysis. Micronutrients below detection limit were defined as suboptimal cellular concentrations according to the analysis laboratory and therefore were included.

### Serum concentrations of micronutrients

For serum micronutrient analysis, blood was collected by venipuncture between 8.00 a.m. and 10.00 a.m. after an overnight fast. Serum was separated by centrifugation at 2,000 x g for 15 min at 4°C. Aliquots were immediately stored at −80°C and sent to an external laboratory on the same day. HPLC was used to assay vitamin A and α-tocopherol. Measurement of vitamin C, calcium and iron was performed by photometry. Furthermore, serum was analyzed for 25(OH)D (radioimmunoassay), zinc and selenium (atomic absorption spectrometry) and vitamin B_12_ and folate (luminescence immunoassay).

### Vitamin C concentrations in leukocytes

Isolation of leucocytes for vitamin C analysis was performed immediately after blood withdrawal. Briefly, sedimentation of erythrocytes was performed in a hermetic tube in dark environment using 6% Dextran according to the method of Denson *et al.*[20]. Supernatant was centrifuged for 10 min at 320 x g. After resuspension with HA buffer lysis of remaining erythrocytes was performed by 5 min incubation with 10 ml ammonium chloride. After centrifugation (10 min, 600 x g), leukocytes were manually counted and pellets frozen at −80 °C until analysis. Determination of total ascorbic acid concentrations in leukocytes was performed by HPLC. In summary, after thawing, leukocyte pellets reducing agent (20 μl 20% TCEP solution [tris (2- carboxyethyl) phosphine hydrochlorid]) was added, thoroughly mixed and incubated on ice for 5 min. For cell lysis 80 μl 10% meta-phosphoric acid was added, mixed and centrifuged (10 min, 13,000 x g, 4°C). Ascorbic acid concentrations in the supernatant were determined by UV/VIS detector at 245 nm.

All samples for vitamin C analysis were rapidly processed and shielded from light exposure.

### Statistical analysis

Values are expressed as means ± SD if not indicated otherwise. For the comparison of dietary record parameters with the reference population, Gaussian distribution was assumed due to sample size (n = 15,371). Differences between the obese study population and the NNSII data were analyzed using unpaired t-tests. The paired *t*-test was applied to compare repeated measures of micronutrients. If Gaussian distribution could not be assumed Wilcoxon signed-rank test was used instead of t-tests. Frequencies were analyzed with cross tables according to the method of McNemar. Analysis of covariance (ANCOVA) was performed to control for the effect of sex, age, energy, and medication use on micronutrient concentrations. Relations between continuous variables were analyzed by calculating the Spearman´s rank correlation coefficient or, in case of normal distribution of the data, the Pearson’s correlation coefficient. P values <0.001 were interpreted as statistically highly significant, p-values between 0.001 and <0.01 as very significant and p-values between 0.01 and <0.05 as significant differences. All analyses were carried out by using the statistics software SPSS, version 19.0 (IBM®SPSS®, Chicago, IL), and Graph Pad Prism, version 5.0 (GraphPad Software, Inc., La Jolla, CA).

## Results

### Baseline characteristics, weight loss and drug intake

Prior to intervention all subjects had at least grade I obesity (BMI ≥ 30 kg/m^2^), about 50% even obesity grade III (BMI ≥ 40 kg/m^2^). The obese study group (dietary records) and the reference population were well matched in respect to age, but the obese group comprised more females (Table 2). BMI of the reference population was significantly lower compared to the obese study group (26.5 kg/m^2^ versus 40.9 kg/m^2^, p < 0.001). 32 out of 104 obese individuals (31%) underwent the LCD intervention. On average, these subjects lost 16.2% of initial body weight during the three-month LCD period. The LCD intervention subgroup (n = 9), which was followed up until program end (12 months after start of intervention), did not differ in weight loss during the LCD period compared with the whole LCD intervention group (Table 3).

**Table 2:** Subjects characteristics of the obese study population (dietary record analysis) and a subgroup undergoing low-calorie diet

	**Reference population (NNSII)**	**Obese study population (n = 104)**	**LCD intervention group (n = 32)**	**LCD intervention subgroup (follow-up until program end) (n = 9)**
Sex (%)				
male	46.2	26.9	12.5	33.3
female	53.8	73.1	87.5	66.7
Age	45.8	45.8 ± 11.0	47.0 ± 10.23	48.9 ± 8.94
Weight [kg]	76.7	117.8 ± 29.85	118.4 ± 19.93	125.5 ± 22.54
BMI [kg/m2]	26.5	40.9 ± 7.20	41.8 ± 7.21	43.0 ± 6.42

Data are presented as mean ± SD.

**Table 3:** Weight loss during low-calorie diet (n = 32) and further follow-up until program end (n = 9)

	**WL after LCD [kg]**	**RWL after LCD [%]**	**WL 12 at program end [kg]**	**RWL at program end [%]**
LCD intervention group (n = 32)	19.5 ± 5.65	16.2 ± 3.24		
LCD intervention subgroup^a^ (n = 9)	21.8 ± 8.41	17.0 ± 4.12	19.9 ± 15.0	15.5 ± 10.6

^a^follow-up until program end.

Abbreviations: *WL* Weight loss, *RWL* Relative weight loss.

86% of the subjects undergoing LCD took medication on a regular basis, which may have resulted in drug-nutrient interaction effects, thus influencing micronutrient bioavailability: ACE inhibitor (21%), proton pump inhibitor (8%), antidepressants (14%), L-thyroxine (14%), biguanide (7%), insulin (7%), loop diuretics (8%), NSAID (7%) and xanthine oxidase inhibitor (14%). Frequencies and dosage of medication decreased slightly in 36% of the cases during the LCD period. Controlling for sex, age, energy intake, and medication use, did not reveal significant effects on micronutrient concentrations.

### Micronutrient intake

Micronutrient intakes in obese subjects are shown in Table 4. Micronutrient intake in female subjects was significantly lower for five micronutrients compared to the reference population, in male subjects for six micronutrients, respectively. Women consumed nine micronutrients in amounts below DRI whereas with men this applied to seven micronutrients. Lowest micronutrient intakes compared to DRI were observed for retinol, ß-carotene, vitamin D, folate, iron and iodine (>75% of the obese study population below DRI) and vitamin E, C and calcium (>50% respectively).

**Table 4:** Micronutrient intake of the obese study population compared to DRI and the reference population

		**Female subjects (n = 76)**					**Male subjects (n = 28)**				
**Micronutrients**		**DRI**^**a**^	**Obese study population**	**Reference population (NNS II)**	***P***^**b**^	**Obese subjects [%] below DRI**	**DRI**^**a**^	**Obese study population**	**Reference population (NNS II)**	***P***^**b**^	**Obese subjects [%] below DRI**
Retinol	[μg]	800	629.0 ± 76.57	600.0 ± 0.01	ns	86	1000	594.2 ± 55.92	1000.0 ± 0.01	<.001	89
β-Carotene	[mg]	4.8	3.0 ± 0.24	5.4 ± 0.04	<.001	86	6	2.7 ± 0.31	5.1 ± 0.04	<.001	93
Vitamin D	[μg]	5	2.5 ± 0.32	2.9 ± 0.03	<.05	87	5	2.4 ± 0.31	3.8 ± 0.04	<.001	93
Vitamin E	[mg]	12	11.7 ± 0.56	13.4 ± 0.08	<.05	55	14	11.4 ± 0.80	16.0 ± 0.12	<.05	75
Vitamin B_1_	[mg]	1	1.2 ± 0.04	1.3 ± 0.01	ns	25	1.2	1.4 ± 0.10	1.8 ± 0.01	<.05	39
Vitamin B_2_	[mg]	1.2	1.4 ± 0.04	1.7 ± 0.01	<.05	33	1.4	1.5 ± 0.09	2.2 ± 0.02	<.01	39
Niacin-equivalent	[mg]	13	31.2 ± 0.98	28.6 ± 0.13	ns	0	16	34.9 ± 1.67	39.8 ± 0.22	ns	0
Vitamin B_6_	[mg]	1.2	1.5 ± 0.04	2.0 ± 0.01	<.001	24	1.5	1.7 ± 0.09	2.6 ± 0.02	<.01	43
Folate-equivalent	[μg]	400	215.2 ± 8.07	290.0 ± 1.94	<.001	99	400	213.4 ± 11.07	338.0 ± 3.09	<.001	100
Vitamin B_12_	[μg]	3	4.9 ± 0.31	4.3 ± 0.02	ns	30	3	5.7 ± 0.36	6.5 ± 0.04	ns	4
Vitamin C	[mg]	100	92.1 ± 5.30	152.0 ± 0.95	<.001	66	100	86.2 ± 8.50	152.0 ± 1.14	<.001	71
Sodium	[g]	550	3321.7 ± 119.01	2494.0 ± 9.41	<.001	0	550	3981.6 ± 246.13	3458.0 ± 15.72	<.05	0
Potassium	[mg]	2000	2649.1 ± 72.92	3243.0 ± 11.29	<.001	14	2000	2757.2 ± 146.67	3789.0 ± 15.06	<.001	11
Calcium	[mg]	1000	979.9 ± 46.72	1019.0 ± 4.33	ns	63	1000	1020.4 ± 75.47	1143.0 ± 5.93	ns	61
Magnesium	[mg]	300	365.2 ± 12.23	376.0 ± 1.23	ns	29	350	380.9 ± 23.01	452.0 ± 1.74	<.05	46
Iron	[mg]	15	12.9 ± 0.36	12.3 ± 0.04	ns	78	10	14.7 ± 0.79	15.2 ± 0.06	ns	7
Zinc	[mg]	7	12.0 ± 0.36	9.5 ± 0.03	<.001	3	10	14.2 ± 0.71	12.3 ± 0.05	<.05	4
Iodine	[μg]	200	83.9 ± 4.08	98.0 ± 0.45	ns	99	200	92.8 ± 6.67	107.0 ± 0.5	ns	100

Abbreviations: DRI = Dietary Reference Intake, NNS II = National Nutrition Survey II.

Data are presented as mean ± SE.

^a^ Recommendations for nutrient intake in Germany, Austria and Switzerland (D-A-CH reference values)[18].

^b^ Obese study population compared to German reference population (NNS II).

Micronutrient intake did not differ between the whole study group (n = 104) and the subset of obese individuals undergoing LCD, which were further subjected to intracellular and serum micronutrient analysis.

### Micronutrient deficiencies in serum before and after LCD

**Figure 1:**
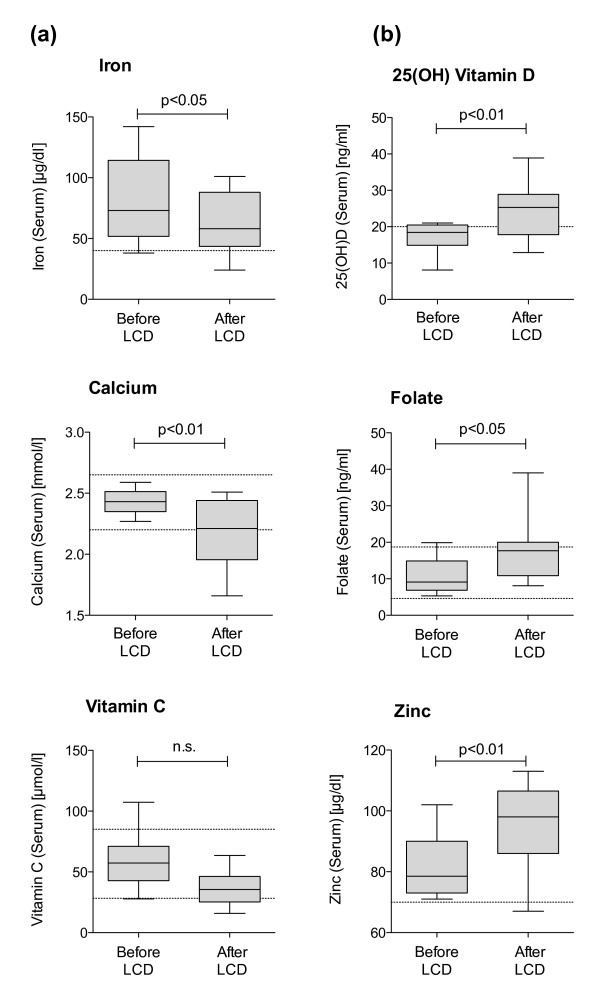
**Serum micronutrients levels that decreased (a) or increased (b) in the course of intervention using a DRI- covering low-calorie formula diet (n = 14).** The dotted lines indicate the reference limits for adequate serum levels. Data are presented as median +/− quartiles (boxes) and 1.5 interquartile ranges (whiskers).

Baseline deficiencies in serum micronutrient concentrations were observed for 25(OH)D, vitamin C, selenium and iron (Table 5). Except for 25(OH)D, the number of cases with deficiencies either remained (selenium, iron, both n.s.) or tended to further increase, which was the case for vitamin C (+15% of subjects with deficiency, n.s.). After the formula period, additional deficiency in zinc (7.7%) and calcium (53.8%) occurred. Figure 1 shows serum concentrations that decreased (calcium (p < 0.01) and iron (p < 0.05)) or increased (25(OH)D (p < 0.01), folate (p < 0.05) and zinc (p < 0.01)), or remained unchanged (vitamin C (n.s.)) in the course of intervention using formula diet.

**Table 5:** Serum micronutrient levels and deficiencies in obese subjects before and after low-calorie diet (n = 14)

		**Before formula diet**		**After formula diet**		
		**Mean ± SD**	**Deficiency [%]**	**Mean ± SD**	**Deficiency [%]**	**p^a^**
Vitamin A	[mg/l]	0.67 ± 0.19	0	0.78 ± 0.22	0	n.s.
Vitamin E	[mg/l]	11.57 ± 4.31	0	12.54 ± 2.66	0	n.s.
25(OH)D	[ng/ml]	17.22 ± 4.02	57.1	24.32 ± 7.25	30.8	<.01
Vitamin C	[mg/l]	52.01 ± 10.65	10.0	43.86 ± 15.96	25.0	n.s.
Vitamin B_12_	[pg/ml]	474.1 ± 155.3	0	528.3 ± 165.3	0	n.s.
Folate	[ng/ml]	10.51 ± 4.7	0	15.42 ± 4.9	0	<.05
Selenium	[μg/l]	87.71 ± 11.74	14.3	95.42 ± 18.67	16.7	<.05
Iron	[μg/dl]	81.50 ± 35.05	14.3	61.62 ± 26.17)	15.4	<.05
Zinc	[μg/dl]	82.14 ± 10.64	0	94.85 ± 13.80	7.7	<.01
Calcium	[mmol/l]	2.44 ± 0.10	0	2.18 ± 0.28	53.8	<.01

^a^ p-values were calculated in relation to absolute values using paired t-tests.

### Micronutrient deficiencies in BMC before and after LCD

**Figure 2:**
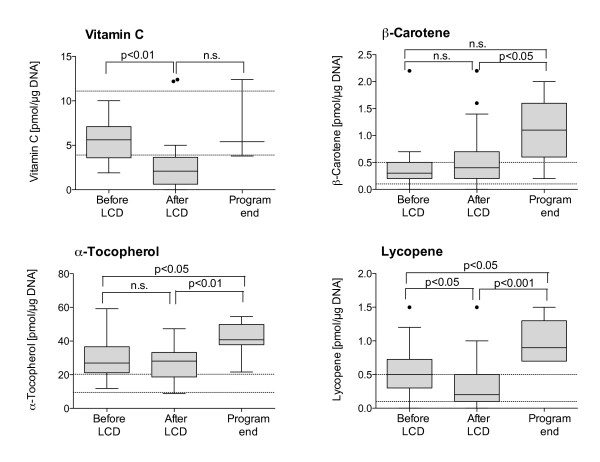
**Intraepithelial micronutrient levels before and after obesity therapy using a low-calorie formula diet (n = 32) and during further follow-up (n = 9).** Samples with no detectable micronutrient levels were excluded in this analysis. Data are presented as median +/− quartiles (boxes) and 1.5 interquartile ranges (whiskers). The dotted lines indicate the reference ranges. Statistical analysis: paired *t*-test.

Intracellular antioxidant status in BMC is displayed in Figure 2. Before intervention, deficiencies were observed for vitamin C (63.3%), ß-carotene (20.0%) and lycopene (10.0%). After the LCD period BMC concentrations of vitamin C (p < 0.05) and lycopene (p < 0.05) decreased, resulting in a higher percentage of subjects with deficiencies (vitamin C +30.2%, p < 0.01 and lycopene +6.1%, n.s.). At the end of the weight loss program (9 months after termination of the formula diet) antioxidative status was optimal or even above reference range, except for vitamin C (deficiencies in 77.8% of subjects), which also tended to improve (n.s.).

### Vitamin C in serum, BMC and leukocytes before and after LCD

**Figure 3:**
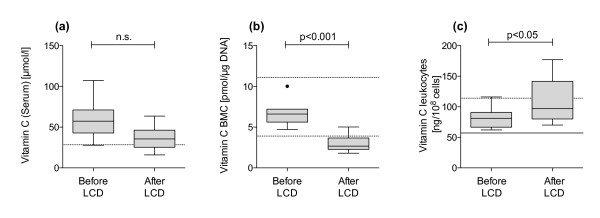
**Comparison of vitamin C levels determined in serum (a) or intracellular, either in buccal epithelial cells (b) or in peripheral blood leukocytes (c), of obese patients who underwent a DRI-covering low-calorie formula diet program (n = 14).** The dotted lines indicate the reference limits for adequate serum levels, the continuous line separates the ’low level range’ from ’manifest deficiency range’ according to reference data from healthy populations. Data are presented as median +/− quartiles (boxes) and 1.5 interquartile ranges (whiskers). BMC samples below detection level were excluded from this analysis.

No correlation between vitamin C serum, BMC and leukocyte concentrations was observed. Serum and BMC vitamin C levels tended to decrease during the formula period (serum 59.44 ± 22.73 μmol/l before intervention, 37.29 ± 13.48 μmol/l after formula diet (n.s.), BMC 6.62 ± 1.52 pmol/μg DNA and 2.95 ± 1.10 pmol/μg DNA (p < 0.001), respectively), whereas leukocyte vitamin C concentrations increased (81.64 ± 16.30 ng/10^8^ cells to 111.2 ± 36.32 ng/10^8^ cells, p < 0.05) (Figure 3).

### Correlations with C-reactive protein (CRP), body weight and body fat

CRP concentrations positively correlated with body weight (r = 0.6558, p < 0.0001). There was a negative correlation between iron and CRP (r = −0.456, p < 0.05). Lipophilic vitamins were negatively associated with body fat in percent of total body weight (25(OH)D: r = −0,6369, p < 0.0001, vitamin A: r = −0,4663, p < 0.05, vitamin E (serum): r = −0,4378, p < 0.05).

## Discussion

The present study clearly confirms previous observations stating that obese individuals are characterized by micronutrient deficiencies [4,6-8,10-13]. The deficiencies are suggested by both low intake and low serum and intracellular levels as shown by our data.

The study demonstrates an insufficient dietary micronutrient supply of retinol, ß-carotene, vitamin D, vitamin E, vitamin C, folate, iron, and calcium in obese individuals. A highly significant difference compared to the reference population, both in men and women, was observed for ß-carotene, folate, vitamin C, and fiber - nutrients which are essentially found in fruits, vegetables and whole-grain products, thus pointing to an unbalanced diet leading to micronutrient deficiencies.

Evaluation of nutritional intake has some methodological weakness such as underreporting that limits the interpretation of dietary record data. The resulting error is possibly even more distinct in obese subjects [21-24]. However, underreporting especially applies to carbohydrate-rich snacks [25], which are low in micronutrient content by nature, and not to ‘healthy’ food. Therefore we assume that this error is negligible regarding micronutrient intake.

Before intervention, obese subjects showed deficiencies of several micronutrients in serum or BMC, namely 25(OH) vitamin D, vitamin C, selenium, iron, ß-carotene and lycopene. We did not find any statistically significant correlation between micronutrient intake and serum or BMC concentrations, which could be due to the limited subject number. However, the reported intakes of ß-carotene, vitamin D, vitamin C, and iron were clearly below DRI and point to a relation between micronutrient intake and body micronutrient status.

Based on our data, we conclude that a DRI-covering low-calorie formula diet does not meet the demands of obese individuals. The reasons can be manifold and could cover metabolic alterations during a period of major weight loss, unbalanced dispersal of lipophilic compounds and fat-tissue specific oxidative stress. Indeed, we observed even more subjects with deficiencies in some micronutrients after a three-month period of formula diet compared to the baseline status before intervention. In particular, vitamin C, selenium, iron, zinc, and lycopene deficiencies increased or could not be corrected by protein-rich formula diet containing vitamins and minerals according to DRI. The formula meal replacement products contained micronutrients in amounts often even higher than those recommended for the general population. We also observed an increase in subjects with calcium deficiency. However, serum calcium concentration is not an adequate measure for dietary calcium intake, but indicates an electrolyte imbalance induced by weight loss and accompanying fluid changes in the body.

Possible interaction effects between pharmaceuticals and micronutrients have to be considered, which may account for increased demands. Within this study, frequency and dosage of drug use did not change during the LCD period in most of the cases and thus most likely did not confound the results. In 36% of the subjects the dosage of at least one drug was slightly reduced, which would, if anything, have improved micronutrient bioavailability.

We did not find statistically significant correlations between vitamin E in serum and BMC, but the decrease in BMC concentrations could point to the fact that intracellular levels respond more sensitively to altered oxidative stress, which particularly occurs in a period of major weight loss [26]. This is explained by an upregulation of the renin-angiotensin system and a reduction of glutathione and glutathione peroxidase in erythrocytes, resulting in higher concentrations of reactive oxygen species which again promote the metabolic syndrome [6]. All three lipophilic vitamins negatively correlated with total body fat, assessed by bioelectric impedance analysis. This is most likely due to the storage capacity for lipophilic compounds, which is so far only suggested for 25(OH)D [27,28]. It is tempting to speculate that this also applies to other lipophilic compounds. Thus, a higher amount of fat tissue could lead to an increase in accumulation of lipophilic vitamins, which in turn are lacking in the serum pool. The positive association between adipose tissue mass and systemic 25(OH)D concentrations, the almost 2fold reduction of 25(OH)D deficiency following LCD, and the significant increase in 25(OH)D serum concentrations observed in our study all suggest storage in adipose tissue and release during weight loss, also described elsewhere [29,30].

McClung *et al.*[31] hypothesized that obesity influences iron absorption by inflammatory mediated mechanisms. Proinflammatory cytokines promote hepcidin release in liver and fat tissue, which is involved in iron homeostasis, inhibiting absorption in enterocytes [31]. This hypothesis is supported by a significantly negative correlation of iron concentration with CRP levels observed in our obese subjects.

Measurement of vitamins in blood samples might not reflect the amount of vitamins absorbed or the concentration in tissues [32,33]. To gain information on body distribution we monitored both serum and intracellular levels of vitamins. BMC are easily available and serve as a model system for assessment of the nutritional and antioxidative status, as well as to control for the success of supplementation and effect of medicamentous therapies [33-35]. With a cellular turnover of 5 to 25 days, BMC are supposed to reflect the current cellular supply [36].

We evaluated the level of vitamin C, lycopene, α-tocopherol, and ß-carotene and thus the antioxidative capacity directly in BMC. These dietary-derived antioxidants are an important component to support the exogenous antioxidative system [33]. Except for ß-carotene and α-tocopherol, which were only slightly reduced, we observed a significant decrease in vitamin C and lycopene during the three-month formula diet suggesting again an enhanced oxidative stress in obese individuals and thus a higher demand of antioxidative vitamins compared to healthy, normal weight subjects, especially in a period of major weight loss.

The current choices for functional markers of vitamin C status are vitamin C concentrations in plasma and leukocytes. Plasma or serum vitamin C levels are highly sensitive to recent dietary intakes, but may not reflect tissue content as well as leukocyte levels [37,38]. Serum vitamin C concentrations remained unchanged during the vitamin C enriched formula diet, whereas leukocyte levels improved. Leukocyte concentrations more reliably display long-term supply and deficiencies [37,38]. Bioavailability of vitamin C is a complex issue involving distribution to the tissues and utilization by the tissues [35]. Before intervention, leukocyte levels were depleted and might replete preferentially due to their high metabolic priority [37]. Contrary to our expectations intracellular vitamin C concentrations in BMC significantly decreased. To obtain a state of complete saturation, the repletion dynamics of vitamin C in certain tissues may be more specific than others when the vitamin intake during repletion is limited [37]. However, the results should be interpreted carefully because of the small sample size, the variation of vitamin C content throughout cell types and the reliability of sampling and analysis procedure due to the unstable nature of vitamin C. In contrast to the fat-soluble antioxidants α-tocopherol, lycopene and ß-carotene, which were demonstrated to be reliable markers in BMC [32,33,35,39,40], the capacity of BMC vitamin C concentrations and its reliability as a biomarker for vitamin C status has not been described so far.

Interestingly, we observed a significant increase in all antioxidants measured in BMC at the end of the program year. This subgroup only included nine subjects, but suggests a change in dietary habits following intensive weight reduction and diet counseling. These subjects were successful in losing weight and were able to maintain this weight after the formula diet, therefore most likely putting their nutritional knowledge into practice. However, this finding needs to be confirmed by other studies.

A potential limitation of the study is that the dietary assessment strategies differed in the two study populations. In the NNSII a comprehensive dietary history method including cross-check features was used. The dietary record approach in this study was chosen to provide quantitatively accurate information on food consumption as well as influence of food processing, but may be biased due to the estimation of the weight of food consumed in some cases instead of weighing, and also by affecting eating behavior. Moreover, for several micronutrients simple blood concentrations were measured, which might not always reflect a complete picture of the nutritional status. At this stage we only included a small sample size, which limits the explanatory power of the study results.

## Conclusions

To the best of our knowledge, this is the first report analyzing if DRI of micronutrients meet the demands of obese individuals consuming a standardized LCD diet. Our pilot study provides evidence that a poor micronutrient status in obesity is not only caused by intakes that are below the DRI. A formula diet providing 100% of micronutrients according to DRI did not cover the demands of some micronutrients in obese subjects. This can be explained by metabolic alterations during a period of major weight loss, unbalanced dispersal of lipophilic compounds and fat-tissue specific oxidative stress. The underlying mechanisms should be further addressed, as well as whether obese individuals receiving an energy-balanced DRI-covering diet also manifest micronutrient deficiencies.

## Competing interest

All authors declare no competing interest; they are all independent from funders. SCB and ADM are part of the Competence network of obesity, which is largely funded by a research grant of the Federal Ministry of Education and Research, Germany; within this network, the research group of SCB is funded in part by Nestlé HealthCare Nutrition GmbH, Munich, Germany. The sponsors had no influence in study design, analysis, and interpretation of data, as well as in the writing of the manuscript. No other relationships or activities exist that could appear to have influenced the submitted work.

## Authors’ contributions

The study was designed by ADM and SCB. ADM and GW carried out the study, collected, and analyzed the data. ADM drafted the manuscript. SCB reviewed the manuscript. All authors read and approved the final manuscript.
